# Proteomic and Transcriptomic Profiling Reveals Mitochondrial Oxidative Phosphorylation as Therapeutic Vulnerability in Androgen Receptor Pathway Active Prostate Tumors

**DOI:** 10.3390/cancers14071739

**Published:** 2022-03-29

**Authors:** Caroline Xue, Eva Corey, Taranjit S. Gujral

**Affiliations:** 1 Division of Human Biology, Fred Hutchinson Cancer Research Center, Seattle, WA 98109, USA; cxue@fredhutch.org; 2 Department of Urology, University of Washington, Seattle, WA 98195, USA; ecorey@uw.edu

**Keywords:** metastatic prostate cancer, patient-derived xenograft, reverse phase protein arrays, gene set expression analysis

## Abstract

**Simple Summary:**

Metastatic prostate cancer (PC) is the second leading cause of cancer deaths in males. The lack of preclinical models and molecular characterization for advanced stage PC is a key barrier in understanding the aggressive subsets androgen receptor (AR) pathway active or AR-null castration-resistant prostate cancers (CRPC). Our study aimed to assess the potential of patient-derived xenograft (PDX) models and an approach integrating proteomic and transcriptomic techniques to explore the underlying drivers of metastatic PC. Transcriptomic and proteomic profiling of 42 PDX prostate tumors uncovered both previously established and unexpected molecular features of aggressive PC subsets. Of these, we confirmed the functional role of mitochondrial metabolism in AR-positive CRPC.

**Abstract:**

Metastatic prostate cancer (PC) is the second leading cause of cancer deaths in males and has limited therapeutic options. The lack of preclinical models for advanced stage PC represents one of the primary barriers in understanding the key genetic drivers of aggressive subsets, including androgen receptor (AR) pathway active and AR-null castration-resistant prostate cancers (CRPC). In our studies, we described a series of LuCaP patient-derived xenograft (PDX) models representing the major genomic and phenotypic features of human disease. To fully exploit the potential of these preclinical models, we carried out a comprehensive transcriptomic and proteomic profiling of 42 LuCaP PDX prostate tumors. The collected proteomic data (~6000 data points) based on 71 antibodies revealed many of the previously known molecular markers associated with AR-positive and AR-null CRPC. Genomic analysis indicated subtype-specific activation of pathways such as Wnt/beta-catenin signaling, mTOR, and oxidative phosphorylation for AR-positive CRPC and upregulation of carbohydrate metabolism and glucose metabolism for AR-null CRPC. Of these, we functionally confirmed the role of mitochondrial metabolism in AR-positive CRPC cell lines. Our data highlight how the integration of transcriptomic and proteomic approaches and PDX systems as preclinical models can potentially map the connectivity of poorly understood signaling pathways in metastatic prostate cancer.

## 1. Introduction

Prostate cancer (PC) is the second leading cause of cancer-related death among men in Western countries [1]. Although agents targeting the androgen receptor (AR) improve the survival of individuals with hormone-dependent PC, most patients ultimately progress toward androgen receptor pathway-negative prostate cancer and further metastatic disease. At least 10% of advanced AR-positive castration-resistant PC (CRPC) transits towards a further aggressive subtype, AR-null CRPC [2]. Despite recent studies identifying genetic and epigenetic regulators of prostate cancer lineage plasticity [3,4], the underlying molecular mechanisms driving these subtypes are still unclear.

The lack of a physiologically relevant preclinical PC model system represents one of the critical barriers to understanding the essential genetic drivers of aggressive subsets. In vitro models, including tumor-derived cell lines and organoids, are commonly used in studies in the PC field on account of their low-cost, large-scale production and ease of experimental setup. However, these 2D cell culture models do not fully recapitulate the complex tumor microenvironment and suffer from poor correlations with in vivo and clinical response. Recently, a series of LuCaP prostate cancer patient-derived xenograft (PDX) tumors were established, representing the major genomic and phenotypic features of the human disease, including amplification of AR, loss of *TP53* and *PTEN*, and showing heterogeneity in responses to treatment of advanced PC [3]. Further, PDX models retain the architecture of and a similar murine stromal component to the original tumor and are therefore considered more accurate representations of the complex tumor microenvironment [5]. To further extend the potential of these preclinical models, comprehensive genomic and proteomic profiling is greatly needed. Here, our objective was to profile signaling pathways that drive metastatic subtypes of PC by integrating proteomic and transcriptomic data for the established PDX models.

## 2. Materials and Methods

### 2.1. LuCaP PDX Samples

A total of 42 PDX tumor samples representing 14 LuCaP PDX models (three independent tumors per model were used to perform unbiased profiling of signaling pathways (Table S1)). The LuCaP PDX tumors were established by subcutaneously implanting patient-derived advanced prostate cancer bits from primary tumors and multiple metastatic sites, including lymph nodes, liver, bladder, and rib, into male SCID mice as described previously [6]. Among 14 LuCaP PDX models, 9 were differentiated adenocarcinomas, 4 were neuroendocrine carcinomas, and 1 was double-negative PC.

### 2.2. Preparation of Tumor Lysates for RPPA

Tumor protein lysates for RPPA were prepared as described previously [7]. Briefly, the snap-frozen LuCaP bits were minced and homogenized in lysis buffer containing 2% sodium dodecyl sulfate (SDS), 50 mM Tris-HCl, 5% glycerol, 5 mM ethylenediaminetetraacetic acid (EDTA), 1 mM NaF, 10 mM b-GP, 1 mM PMSF, 1 mM Na_3_VO_4_, and 1 mM dithiothreitol (DTT) and supplemented with protease and phosphatase inhibitor cocktail (Thermo Scientific, Waltham, MA, USA). Protein lysates were filter cleared using AcroPrep™ Advance 96-Well Filter Plates (Pall, New York, NY, USA) by centrifuging at 1962 g for 4–6 h at room temperature. The total protein amount in tumor lysates was quantified using a BCA protein assay kit (Thermo Scientific, MA, USA) according to the manufacturer’s instructions.

### 2.3. Reverse Phase Protein Array Construction

Protein microarrays were printed and processed as described in detail previously [8]. Tumor protein lysates were printed onto 16-pad nitrocellulose-coated slides (Grace Biolabs, Bend, OR, USA) using an Aushon 2470 microarrayer (Aushon BioSystems, Billerica, MA, USA). Each sample was printed in duplicate, generating 84 sample spots on each subarray. A total of 6 slides were printed, allowing probings with 96 validated antibodies (Table S2). Slides were stored at −20 °C until processing.

### 2.4. Array Processing and Probing

RPPA slides were washed with 1 M Tris-HCl (pH 9.0) for 2–4 days to remove SDS. Slides were then washed 2–3 times with phosphate-buffered saline (PBS) for 5 min each and blocked with Odyssey Blocking Buffer (OBB, Licor, NE, USA) for one hour at RT. After blocking, arrays were incubated with primary antibodies in OBB at 4 °C overnight. The next day, arrays were washed thrice with PBS and incubated with IRDye-labeled secondary antibodies in OBB for 1 h at room temperature. Arrays were rewashed thrice in PBS and once in ddH_2_O and spun dry.

### 2.5. Signal Quantification and Data Analysis

The RPPA slides treated with IR-labeled secondary antibodies were scanned using a Licor Odyssey CLX Scanner (LiCOR, Lincoln, NE, USA). Each spot’s total signal intensity was quantified using the Array-Pro analyzer software package (Media Cybernetics, Rockville, MD, USA). The measurement of a specific protein from an individual sample was then normalized to total beta-actin (Sigma, St. Louis, MO, USA, cat. no. A1978).

### 2.6. Cell Lines and Reagents

Prostate cancer cell lines 22RV1, DU145, NCI-H660 cell lines were obtained from the American Type Culture Collection (ATCC) and cultured according to the ATCC culture methods. C4-2B cells were obtained from Dr. Nelson (Fred Hutchinson Cancer Research Center, Seattle, WA, USA). Cells from the 22RV1 and C4-2B lines were maintained in RPMI-1640 (Life Technologies, Grand Island, NY, USA) supplemented with 10% fetal bovine serum (FBS) (Corning, Corning, NY, USA) and 1% penicillin/streptomycin (P/S, 10,000 U/mL) (Life Technologies, NY, USA). DU145 was maintained in DMEM (Corning, NY, USA) supplemented with 10% FBS and 1% P/S. NCI-H660 cells were grown in RPMI-1640 supplemented with 10% FBS, 1% P/S, 1% sodium pyruvate, 4 mM L-glutamine, 0.05% bovine insulin, 10 nM hydrocortisone, 10 nM b-estradiol, 30 nM sodium selenite, and 1% transferrin. Small molecule inhibitors targeting mTOR (Torin 2), oxidative phosphorylation (antimycin), and histone demethylation (GSK-J4) were purchased from Selleckchem (Houston, TX, USA).

### 2.7. Cell Viability Assay

The effects of pathway-specific inhibitors on the viability of PC cells were measured using both live-cell imaging (Incucyte Zoom, Ann Arbor, MI, USA) and CellTiter-Glo assay (Promega, Madison, WI, USA) as described previously [5]. Briefly, PC cells (5 × 10^3^ in 100 µL culture medium) were seeded on a 96-well plate (Corning, NY, USA). The next day, cells were treated with various inhibitors ranging from 100 µM–10 nM in serial dilution of 1/3 and placed in IncuCyte (Essen Biosciences, Ann Arbor, MI, USA) for imaging every 2 h. After 4 days, cells were incubated with CTG reagent for 5 min, and total viability was measured by obtaining luminescent signal intensity. The quantified data were normalized to untreated controls and plotted in Prism (Graphpad Software, San Diego, CA, USA).

### 2.8. Quantitative Western Blotting

Prostate cancer cells were rinsed twice with PBS and lysed in SDS lysis buffer (described above). Protein lysates were filter cleared, and protein concentrations were determined using BCA assay kit and subjected to immunoblotting using standard procedures. For quantitative immunoblots, primary antibodies were detected using IRDye 680-labeled goat anti-rabbit IgG or IRDye 780-labeled goat anti-mouse IgG (LiCOR) at 1:1000 dilution. Bands were visualized and quantified using an Odyssey CLX Scanner (LiCOR).

### 2.9. Gene set Enrichment Analysis (GSEA)

GSEA was performed using the LuCaP prostate cancer PDX gene expression dataset (GEO: GSE93812) [9].

### 2.10. Enrichr and PathwayNet

Enrichr and Pathwaynet analyses were performed using the list of differently expressed genes identified by RPPA result with a cutoff of 1.5-fold between AR-null and AR-positive to identify five genes in AR-null and 11 genes in AR-positive CRPC [10,11].

### 2.11. Preparation of LuCaP Tumor Organotypic Slices

LuCaP tumor slices were prepared as described previously [12]. Briefly, dissected PDX tumor tissues were molded into a 6 mm core using a biopsy punch and used to generate 250 µm slices using a Leica Vibratome VT1200 (Leica). Slices were immediately placed on inserts in 24-well plates and incubated with Williams’ Medium containing 12 mM nicotinamide, 150 nM ascorbic acid, 2.25 mg/mL sodium bicarbonate, 20 mM HEPES, 50 mg/mL of additional glucose, 1 mM sodium pyruvate, 2 mM L-glutamine, 1% (*v*/*v*) ITS, 20 ng/mL EGF, 40 IU/mL penicillin and 40 µg/mL streptomycin. After 48 h, slices were treated with drugs at varying concentrations. Overall tumor tissue viability was measured using RealTime Glo (Promega) reagent according to the manufacturer’s instructions. IVIS images were taken before (day 0) and after drug treatment (day 6) using an IVIS Spectrum instrument (Perkin Elmer, Waltham, MA, USA).

## 3. Results

LuCaP PDX tumors developed from a series of prostate cancer metastases are well-established model systems representing several phenotypic characteristics of clinical PC, including differential androgen receptor activity [6,13,14]. To date, many studies have described histopathological characteristics, pharmacological response, and transcriptomic and genomic features of LuCaP PDX tumors [6,13,14]. However, understanding how the wiring of networks is altered in the regulation of metastatic PC at the level of protein activity is necessary.

### 3.1. Proteomic Profiling of Signaling Pathways in LuCaP PDX Tumors

**Figure 1:**
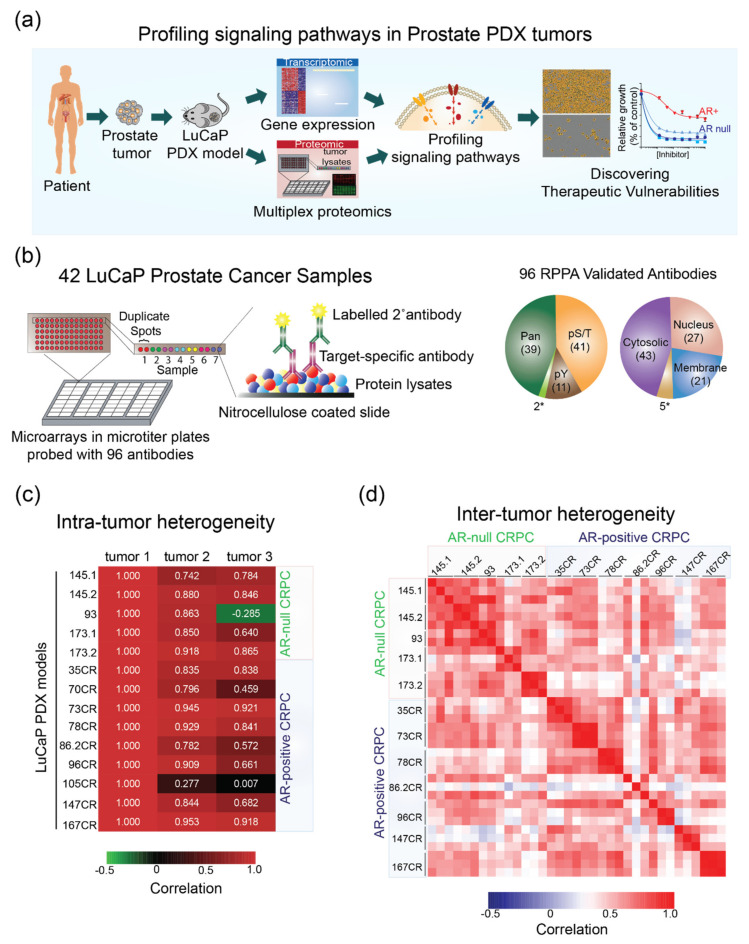
Proteomic profiling of signaling pathways in LuCaP PDX tumors shows a high degree of intra-tumor and inter-tumor correlation. (**a**) A schematic showing the overall goal and design of our study to integrate proteomic and transcriptomic profiling in LuCaP PDX tumors. The resulting state of signaling pathways is then validated in model cell lines and PDX tumors using pathway-specific inhibitors. (**b**) A schematic illustrating RPPA and properties of validated antibodies used in this study. Right: pie charts showing the distribution of all antibodies tested and those yielding positive signals broken down by different targets, PTMs, and subcellular localization. * refers to antibodies detecting cleaved caspases. (**c**) A heatmap showing the correlation between three different tumor samples from each LuCaP PDX model. Sample tumor 1 is used as a reference, and the numbers indicate Pearson correlation. (**d**) A heatmap showing a strong positive correlation within AR-null and AR-positive LuCaP PDX models.

We sought to elucidate critical features of the oncogenic state at the level of protein activities in 42 PDX models established from metastatic PC patient tumors using RPPA (Figure 1a, Table S1). RPPA is a high-throughput immune-based assay that enables multiplexing by printing multiple copies of the same array. Each microarray in the form of a glass slide is coated with 16 separate nitrocellulose membrane grids and can accommodate thousands of lysate samples. The printed slides are then incubated with validated primary antibodies and labeled secondary antibodies to measure the level of protein abundance or post-translationally modified proteins (Figure 1b). Previously, we applied RPPA technology to dissect signaling pathways both in cultured cells and in a limited number of clinical specimens [7,15,16]. Here, we generated approximately 100 arrays using 10 µg of total lysate derived from 42 LuCaP PDX tumor samples. All antibodies used in this study have been vigorously validated for specificity using conventional Western blotting and used in RPPA [8,16] (Figure 1c). These antibodies represent key signaling proteins spanning a broad range of signaling pathways (Table S2). Of these, 60% of antibodies (57 out of 96) were directed against post-translational modification of proteins (Figure 1b). The distribution of subcellular localization of proteins targeted by these antibodies is also shown in Figure 1b. This resulted in a set of >4600 proteomic data points based on 55 antibodies that were used on 42 LuCaP PDX samples.

Genomic, epigenetic, transcriptomic, and signal transduction alteration, or clonal evolution can give rise to intra-tumoral heterogeneity [17]. Since we measured signaling profiles in at least three distinct tumors from each LuCaP model, we asked if there is a correlation between different tumors derived from the same model. We observed a high degree of intra-tumor correlation (r > 0.8) in 85% (12 out of 14 models) of overall LuCaP models tested (Figure 1c). One of the tumor samples in LuCaP 93 and two in LuCaP 105 CR and 70 CR models showed poor correlation with other tumor samples within the same model, which could be caused by differential contents of stroma in those samples. Therefore, these samples were omitted from further analysis of inter-tumor heterogeneity data. Overall, high dimensional, proteomic profiling using RPPA produced a high correlation among tumors within the same model.

Intertumoral heterogeneity refers to the combination of intrinsic and extrinsic heterogeneity between patients with tumors of the same histopathological subtype. Of 35 LuCaP PDX samples analyzed in this study, 11 tumors represented AR-null, and 21 represented the adenocarcinoma AR-positive CRPC subtype. Similar to the results from intra-heterogeneity data, we observed a high correlation (*p* > 0.74) among the AR-positive and AR-null PC subtypes of LuCaP PDX tumors, respectively (Figure 1d). The majority of the samples correlated positively (*p* > 0.7) within each subtype of PC and showed moderate-to-high correlation across different subtypes between AR-positive and AR-null (*p* > 0.4). In general, the correlation among AR-positive tumor samples was lower than in AR-null tumors, indicating the heterogeneous nature of the AR-positive population, similarly as observed in a recent study using AR-positive biopsies [18]. These data show similar inter-tumor and intra-tumor heterogeneity in the LuCaP PDX samples to the heterogeneity observed in the clinical samples.

### 3.2. AR-Positive and AR-Null LuCaP PDX Tumors Exhibit Characteristics of Clinical Prostate Carcinoma at the Protein Level

**Figure 2:**
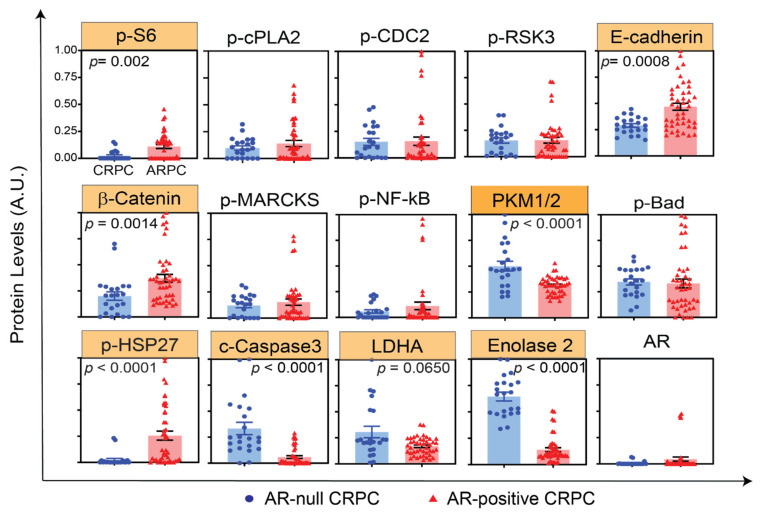
RPPA profiling of LuCaP PDX tumors identifies previously known markers of AR-null and AR-positive CRPC. Representative plots showing 12 different proteomic measurements in 22 AR-null (blue) and 44 AR-positive (red) tumor lysate samples. The proteins highlighted in yellow indicate statistically significant results (*p* < 0.02).

Previous studies have identified several pathways activated in AR-positive CRPC, including PI3K/Akt, Notch, and DNA damage response and predominantly activated regulators of AR-null CRPC, including SRRM4, AURKA, and MYCN, as well as epigenetic modifiers, such as EZH2 [19,20]. Here, we performed an unbiased screening of oncogenic signaling pathways in LuCaP PDX models using RPPA. Figure 2 shows the state of 15 representative protein signals in AR-null and AR-positive LuCaP PDX tumors, recapitulating many of the previously known markers associated with AR-positive and AR-null subtypes of PC. Corroborating previous reports [21,22,23], our data showed markedly higher levels of Enolase 2 in AR-null (*p* < 0.05, 4.5-fold) and significantly higher levels of phospho-S6 ribosomal protein, β-catenin, E-cadherin, and phospho-HSP27 in AR-positive (*p* < 0.05, >1.5 fold) LuCaP PDX tumors (Figure 2). Interestingly, we also discovered increased levels of proteins involved in glycolysis, including LDHA (2-fold, *p* = 0.0650) and PKM1/2 (1.6-fold, *p* < 0.0001) in AR-null LuCaP PDX tumors (Figure 2), suggesting alterations in metabolic pathways associated with AR-null progression from the adenocarcinoma phenotype. Taken together, consistent changes in the levels of proteins and their activities in LuCaP PDX samples, as observed and previously known markers of PC, provide further evidence that LuCaP samples represent physiologically relevant model systems to interrogate various subtypes of PC.

### 3.3. AR-Positive and AR-Null LuCaP PDX Tumors Display Distinct Signaling Network Topologies

**Figure 3:**
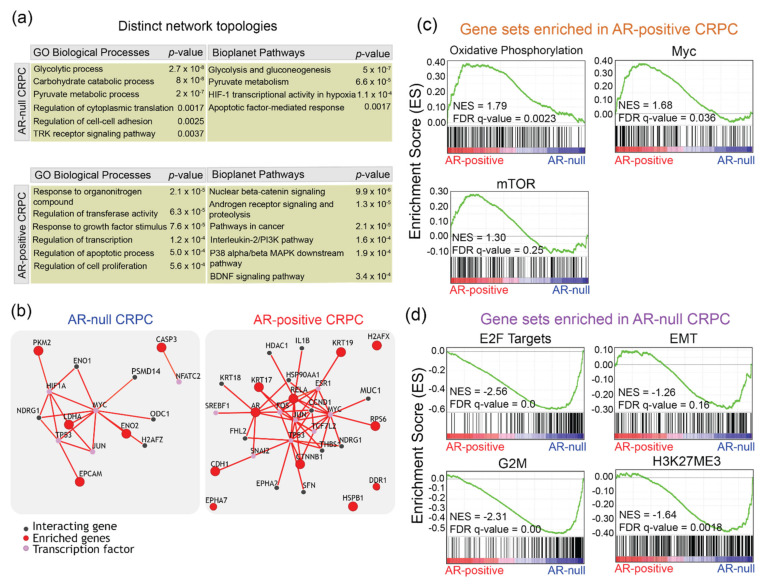
AR-null and AR-positive LuCaP PDX tumors display distinct signaling network topologies. (**a**) Representative tables of enriched pathways and ontologies highlighted by Enrichr analysis with an input of enriched genes that increased by at least 1.5-fold comparatively between AR-positive and AR-null CRPC, using the RPPA proteomic data. (**b**) PathwayNet predicted interactions between the enriched genes and other genes, and transcription factors are shown in network diagrams for AR-null and AR-positive CRPC. (**c**) GSEA enrichment plots showing upregulation of oxidative phosphorylation, c-Myc, and mTOR in AR-positive LuCaP PDX tumors. (**d**) GSEA enrichment plots showing upregulation of E2F targets, EMT, G2M, and H3K27ME3 in AR-null LuCaP PDX tumors.

RPPA data provided an unbiased means to measure levels and activities of the proteins spanning many different signaling pathways. To explore the enrichment of specific molecular pathways associated with AR-positive and AR-null subtypes, we performed unsupervised clustering of proteomic data followed by enrichment analysis using Enrichr. Enrichr analysis was performed using a 1.5-fold cutoff to identify five genes in AR_null and 11 genes in AR-positive [10]. Figure 3a shows representative pathway and ontology enrichment tables for AR-null and AR-positive. Enrichr revealed enrichment of the carbohydrate catabolic process (*p* = 8.0 × 10^−8^), glycolysis and gluconeogenesis (5.0 × 10^−7^), pyruvate metabolism (6.6 × 10^−5^), and HIF-1 transcriptional activity in hypoxia (1.1 × 10^−4^) in AR-null (Tables S3 and S4). Conversely, Enrichr revealed the enrichment of nuclear beta-catenin signaling (*p* = 9.9 × 10^−6^), androgen receptor signaling and proteolysis (1.3 × 10^−5^), the interleukin-2/PI3K pathway (*p* = 1.6 × 10^−5^), and the p38/beta MAPK downstream pathway (1.9 × 10^−4^) (Figure 3a) (Tables S5 and S6). Overall, our proteomic profiling in LuCaP PDX models highlights the activation of many previously known signaling pathways and identified new potential regulators of advanced-stage PC.

To explore the potential interactions between the enriched genes that underlie the tumorigenesis and progression of AR-null and AR-positive and substantiate our Enrichr results, we performed PathwayNet analysis to predict the interactions of the enriched genes discovered through RPPA [11]. Figure 3b displays network diagrams of the predicted interactions of the enriched genes with transcription factors and other genes in AR-null and AR-positive. PathwayNet reveals interactions with MYC, p53, and HIF-1 transcription factors. Of these, the overexpression of MYC has been highlighted as a common feature of AR-null CRPC and an important driver of AR-null CRPC progression in previous studies [4,24]. The predicted interactions with HIF-1 and p53 transcriptional factors are consistent with the Enrichr results, which revealed enriched HIF-1 transcriptional activity in hypoxia and apoptotic factor-mediated response pathways (Figure 3a). Similarly, PathwayNet predicted interactions with MYC, p53, and JUN in AR-positive CRPC (Figure 3b). Interactions with TCF7L2 and FOS transcription factors were also predicted in AR-positive CRPC. Activating protein-1 (AP-1) Jun and FOS have been implicated in PC through interactions with AR signaling [25,26]. The predicted interaction with the TCF7L2 transcription factor, as a critical member of the Wnt/beta-catenin pathway, is consistent with our Enrichr results.

To further substantiate our proteomic data-driven discovery of subtype-specific signaling pathways, we sought to identify a set of genes that are differentially expressed between the subtypes of PC. We performed gene set enrichment analysis (GSEA) using previously determined array CGH data [6]. GSEA revealed enrichment of several pathways and processes, such as androgen response (NES, 2.6; FDR, q-val 0), Myc (NES, 1.7; FDR q-val, 0.03), PI3K-Akt-mTOR (NES, 1.3; FDR q-val, 0.03), and oxidative phosphorylation (NES, 1.8; FDR q-val, 0.002) in AR-positive LuCaP PDX tumors (Figure 3c). Of these, upregulation of c-Myc and activation of PI3K-Akt-mTOR pathways have been identified in human AR-positive tumors [27,28]. These data are also consistent with RPPA profiling which revealed increased phosphorylation of S6 ribosomal proteins (a downstream target of mTOR signaling) in AR-positive LuCaP PDX tumors (Figure 2). More importantly, we identified significant enrichment of genes in the oxidative phosphorylation in AR-positive LuCaP PDX tumors, implicating a role of altered mitochondrial metabolism in the progression of metastatic AR-positive CRPC.

GSEA analysis of AR-null LuCaP PDX tumors revealed enrichment of E2F targets (NES, 2.5; FDR q-val, 0.0), epithelial–mesenchymal transition (EMT) (NES, 1.2; FDR q-val, 0.16), G2M (NES, 2.3; FDR q-val, 0.0) and histone methylation (NES, 1.6; FDR q-val, 0.00) gene sets (Figure 3d). All of these have been previously shown to be associated with the CRPC phenotype in clinical specimens [29,30,31], underscoring that LuCaP PDX tumors mimic features of clinical PC. Together, these data suggest distinct signaling pathways underlying AR-positive and AR-null subtypes.

### 3.4. Discovering Subtype-Specific Therapeutic Vulnerabilities in PC Cell Lines

**Figure 4:**
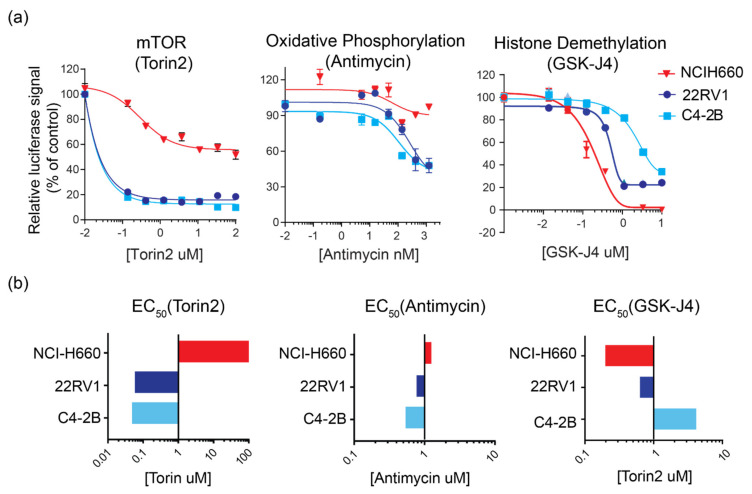
Perturbation studies confirm subtype-specific therapeutic vulnerabilities in prostate cancer cell lines. (**a**) Dose-response curves showing the effect of Torin2 (mTOR inhibitor), antimycin (oxidative phosphorylation inhibitor), and GSK-J4 (histone demethylase inhibitor) on the viability of AR-null and AR-positive cell lines. *n* = at least three biological replicates. (**b**) Bar graphs summarizing the effective drug concentration 50% (EC_50_) of Torin2, antimycin, and GSK-J4 measured in the PC cell lines.

Next, we asked whether the pathways identified by our integrated approach are functionally essential for the growth and survival of PC. We measured cell growth and viability of a panel of AR-positive (22RV1, C4-2B) and AR-null (NCI-H660) cell lines treated with small molecule inhibitors targeting mTOR (Torin2), oxidative phosphorylation (antimycin), and histone demethylation (GSK-J4) pathways. Torin2 is a potent inhibitor of mTOR, ATM, and ATR and has been previously shown to inhibit the growth of several different cancers [32]. Antimycin, a mitochondrial electron transport chain inhibitor, has been shown to prevent the growth of lung cancer cells [33]. GSK-J4 is an inhibitor of several histone demethylases and has been proposed as a potential cancer therapeutic agent [34]. We found that all AR-positive cell lines tested were more sensitive to inhibition of mTOR (EC_50_ < 100 nM) relative to the NCI-H660 cell line (EC_50_ > 100 µM) (Figure 4). Further, inhibition of mitochondrial metabolism significantly decreased the growth of all CRPC cell lines (EC_50_ 160–760 nM) while it had no effect on the growth of NCI-H660 cells (EC_50_ > 1000 nM), suggesting that the mitochondrial metabolism pathway is critical for AR-positive CRPC growth. Conversely, we found that NCI-H660 were more susceptible to inhibition of histone demethylase (EC_50_ < 200 nM) relative to all AR-positive cell lines tested (EC_50_ 550–3500 nM), supporting epigenetic modulators as potential targets for the treatment of the CRPC subtype. Overall, these perturbation studies confirmed the functional importance of distinct pathways identified in AR-positive and AR-null prostate cancers.

### 3.5. Mitochondrial Metabolism as a Potential Target for Metastatic AR-positive CRPC

**Figure 5:**
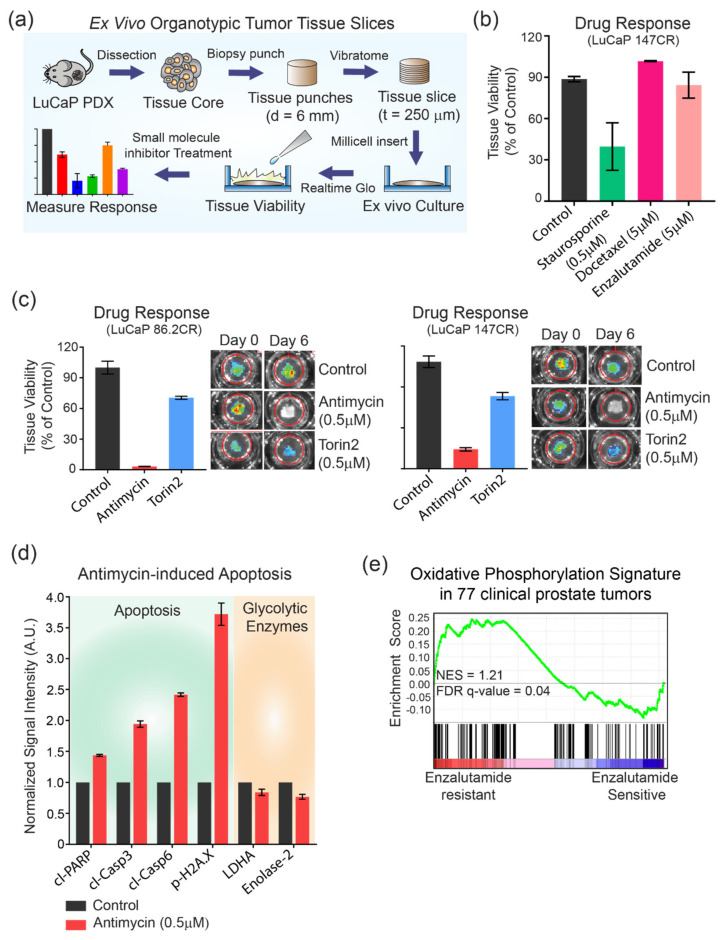
Targeting mitochondrial oxidative phosphorylation inhibits LuCaP PDX tumor viability. (**a**) A schematic showing the workflow of LuCaP PDX tissue slice culture. LuCaP tumor core is punched using a 6 mm biopsy punch and sliced into 250 µm sections using an automated vibratome. The sliced tissues are placed on an insert for culture before tissue viability using RealTime Glo and small-molecule inhibitor treatment. The treated tissue slices are then imaged using IVIS spectrum to measure their response to treatment by acquiring the intensity of bioluminescence. (**b**) A bar graph showing the viability of LuCaP 147 CR tissues treated with staurosporine (0.5 µM), docetaxel (5 µM), and enzalutamide (5 µM). Bars represent the mean of two independent slices. Error bars represent SEM. (**c**) Bar graphs showing the response of LuCaP 86.2 CR and 147 CR to antimycin (0.5 µM) and torin2 (0.5 µM) relative to control. Images of live tissues taken using IVIS Spectrum on day 0 and 6 are shown on the right. (**d**) A bar graph illustrating the signal intensity of proteins associated with apoptosis (green panel) and glycolysis (orange panel) in LuCaP 86.2 CR after treatment with antimycin (0.5 µM). *n* = at least two biological replicates (**e**) A GSEA plot showing enrichment of oxidative phosphorylation gene sets in enzalutamide-resistant prostate cancers.

Having discovered an enrichment of genes involved in oxidative phosphorylation in AR-positive CRPC (Figure 3c) and having shown the potent response of AR-positive cell lines to inhibition of mitochondrial complexes (Figure 4) in 2D culture, we asked whether mitochondrial oxidative phosphorylation is also important for the viability of LuCaP AR-positive PDX tumors. We prepared organotypic tumor slices from two independent PDX tumors (86.2 CR and 147 CR) (Figure 5a and Figure S1). Organotypic tumor slices maintain native tissue architecture and serve as physiologically relevant models for ex vivo biochemical and pharmacological studies [35,36]. Many tumor slices can be prepared from the same tumor core, enabling consistent and higher throughput for downstream studies. Treatment of tumor slices prepared from AR-positive (LuCaP 147 CR) tumors with various pharmacological agents, including high doses (5 µM) of docetaxel and enzalutamide, showed no effect (101% of control for docetaxel and 84% of control for enzalutamide) on tissue viability, while treatment with a low dose (500 nM) of staurosporine (a multi-kinase inhibitor) decreased (>2-fold) overall tissue viability (Figure 5b). This is consistent with our previous study in which LuCaP 147 CR showed negligible response to docetaxel in vivo, suggesting that tumor slices can be used as ex vivo models for pharmacological profiling [6]. Next, we treated tumor slices prepared from two different AR-positive LuCaP models to mTOR inhibitors and mitochondrial metabolism. In both models, antimycin-mediated inhibition of oxidative phosphorylation significantly decreased tumor viability (20-fold in LuCaP 86.2 CR, *p* < 0.005, and 3-fold in LuCaP 147 CR, *p* < 0.005) (Figure 5c). Inhibition of mTOR pathways had a low–moderate effect on overall tumor tissue viability in both models (76% of controls in LuCaP 86.2 CR and 82% of controls in LuCaP 147 CR). To corroborate tumor viability data, we assessed the activation of apoptosis in AR-positive CRPC slices using RPPA. We found a significant increase in the levels of several apoptotic markers, including cleaved caspase-3 (2-fold), cleaved caspase-6 (2.4-fold), cleaved PARP (1.4-fold), and phosphorylation of Histone 2AX (3.7-fold), indicating a commitment to apoptosis in response to antimycin treatment (Figure 5d). However, no changes in the level of LDHA or Enolase2 were observed (Figure 5d). Finally, we show that the oxidative phosphorylation gene signature is significantly enriched in clinical enzalutamide-resistant prostate cancers (NES, 1.25; FDR q-value, 0.04) [37] (Figure 5e). Together, these data show that inhibition of oxidative phosphorylation decreases the growth and viability of AR-positive cell lines and PDX tumors, suggesting mitochondrial metabolism as a potential target for treating metastatic AR-positive CRPC. Furthermore, our data establish that organotypic tumor slices from LuCaP PDX can be used for ex vivo biochemical and pharmacological studies.

## 4. Discussion

Due to the highly unstable and evolving nature of prostate cancer, most patients ultimately become unresponsive even to ‘next-generation’ inhibitors of AR signaling [38]. In particular, subtypes such as advanced stage AR-null are resistant to enzalutamide treatment. Some key molecular drivers of these subtype differentiations include loss of RB1 and TP53 tumor suppressors, activation of BRN2 or mTOR, and alterations to the epigenetic landscape. However, unbiased proteomic studies of prostate cancer progression are lacking. Understanding the signaling landscapes that underlie various advanced-stage prostate cancers will aid the development of much-needed therapeutic agents.

In this study, we utilized LuCaP PDX tumor models, which represent physiologically relevant environments (in contrast to cell lines) to carry out proteomic profiling to identify subtype-specific pathways that are functionally important for advanced stages of PC. Our proteomic data were concordant with results of previously identified molecular markers and networks associated with AR-positive and AR-null subtypes (Figure 2). Consistent with previous reports [16,17,18], we found markedly higher levels of Enolase 2 in AR-null and significantly higher levels of phospho-S6 ribosomal protein, β-catenin, E-cadherin, and phospho-HSP27 in AR-positive LuCaP PDX tumors (Figure 2), validating that LuCaP samples represent a physiologically relevant model system to interrogate various subtypes of PC.

Enrichr identified many known signaling nodes and pathways involved in AR-positive and AR-null subtypes. These include pyruvate metabolism, HIF-1 transcription factor network, glycolysis in AR-null, and Wnt/beta-catenin signaling and AR signaling in AR-positive (Figure 3a). Elevated glucose metabolism, as an important metabolic feature of AR-null and a potential therapeutic target, has been corroborated by previous studies [39,40]. Recent studies have also highlighted the role of hypoxic conditions underlying the development of the AR-null phenotype [41,42]. Further, recent studies have highlighted the enrichment of beta-catenin and implicated the Wnt/beta-catenin pathway with anti-androgen resistance, specifically due to the crosstalk with androgen receptor signaling [37,43]. Other studies have highlighted the role of PI3K/AKT, MAPK, Wnt, and AR signaling crosstalk in prostate tumorigenesis and progression [44,45]. PathwayNet highlighted the role of c-MYC and AP-1 transcription factors in both AR-positive and AR-nul while corroborating Enrichr results (Figure 3b). Through GSEA, we have identified the role of mTOR and oxidative phosphorylation in AR-positive (Figure 3c). Many of these pathways are targets for ongoing clinical trials, underscoring their functional importance in aggressive prostate cancers [46]. We functionally validated the role of oxidative phosphorylation in a panel of AR-positive cell lines and LuCaP tumor tissues, suggesting a metabolic shift towards mitochondrial metabolism associated with an aggressive form of AR-positive (Figure 4 and Figure 5). Furthermore, we have shown the enrichment of oxidative phosphorylation signatures in clinical enzalutamide-resistant prostate cancers (Figure 5e), emphasizing the use of LuCaP models as tools for novel biological discovery.

Recently, metabolic analysis of docetaxel-resistant prostate cancer cells showed a heightened respiratory phenotype and a shift from “Warburg” to oxidative phosphorylation, in agreement with our data [47]. Consistently, mitochondrial bioenergetics also play a significant role in the motility and invasiveness of androgen-independent prostate cancer cells [48]. Notably, a recent study identified higher levels of mitochondrial malate dehydrogenase, MDH2, in AR-responsive prostate cancer [49]. Many previous studies have provided compelling evidence that oxidative phosphorylation remains not only an essential source of ATP for tumors but may also affect important hallmarks, including EMT and resistance to therapy [48,50]. Targeting mitochondrial metabolism inhibits the growth of leukemic stem cells in vitro and in vivo [51]. Together, our discovery of enriched gene sets and data showing the functional importance of oxidative phosphorylation in AR-positive CRPC warrants further investigations focusing on delineating mitochondrial dynamics and bioenergetics in AR-positive CRPC.

LuCaP PDX series represent histopathological, genomic, and proteomic features of clinical prostate cancers, making them suitable tools for biological discovery in the context of prostate cancer progression and drug resistance. However, a major challenge of working with LuCaP PDX models is that the expansion of human tumors under mouse physiological conditions can take months or years, which limits their potential for diagnostic and therapeutic applications. We have shown that organotypic tumor slices can be generated from LuCaP PDX tumors which maintain the characteristic of the native tumor tissue (Figure 5). Tumor slices are not affected by growth selection bias and can be used for short-term assays that could predict clinical drug responses, making this an ideal approach for personalized medicine [52]. We show that ex vivo tumor slices prepared from LuCaP PDX tumors reproduce in vivo responses to docetaxel and enzalutamide (Figure 5). Using this approach, we showed that inhibition of oxidative phosphorylation significantly reduced tumor viability and caused apoptosis in two independent AR-positive LuCaP models (Figure 5), suggesting mitochondrial metabolism as a therapeutic vulnerability in AR-positive CRPC. Overall, we established a new application of LuCaP PDX tumor slices, enabling higher throughput and faster preclinical efficacy studies and potentially affording clinically actionable information for personalized medicine.

## 5. Conclusions

Overall, our comprehensive profiling of signaling pathways in metastatic LuCaP PDX models provides a valuable resource for the prostate cancer community interested in using these preclinical models for their studies. Our results also provide an impetus to other investigators and drug companies to utilize phosphorylation-rich RPPA data for predicting drug responses. Moreover, the integration of transcriptomic and proteomic approaches has the potential to map the connectivity of poorly understood signaling pathways of metastatic prostate cancer.

## Data Availability

The data presented in this study are available in the Supplementary Materials.

## Supplementary Materials

The following are available online at https://www.mdpi.com/article/10.3390/cancers14071739/s1, Figure S1: Characteristics of 42 LuCAP PDX tumor samples, Table S1: Characteristics of 42 LuCAP PDX tumor samples, Table S2: List of validated antibodies used for RPPA analysis, Table S3: Top 25 Enrichr GO Biological Processes in AR-null CRPC, Table S4: Top 25 Enrichr Bioplanet in AR-null CRPC, Table S5: Top 25 Enrichr GO Biological Processes in AR-positive CRPC, Table S6: Top 25 Enrichr Bioplant in AR-positive CRPC.
