# Supplementary material for: Proteomic and Transcriptomic Profiling Reveals Mitochondrial Oxidative Phosphorylation as Therapeutic Vulnerability in Androgen Receptor Pathway Active Prostate Tumors
Source: Cancers (Basel). 2022 Mar 29;14(7):1739. doi: 10.3390/cancers14071739 (PMC8997167; doi:10.3390/cancers14071739)
Supplement: Supplementary file 1 [file cancers-14-01739-s001.zip › cancers-1565329-supplementary.pdf]

Supplementary Materials

Proteomic and Genomic Profiling Reveals Mitochondrial Oxidative Phosphorylation as Therapeutic Vulnerability in Castrate-Resistant Prostate Tumors

Caroline Xue, Eva Corey, and Taranjit S Gujral

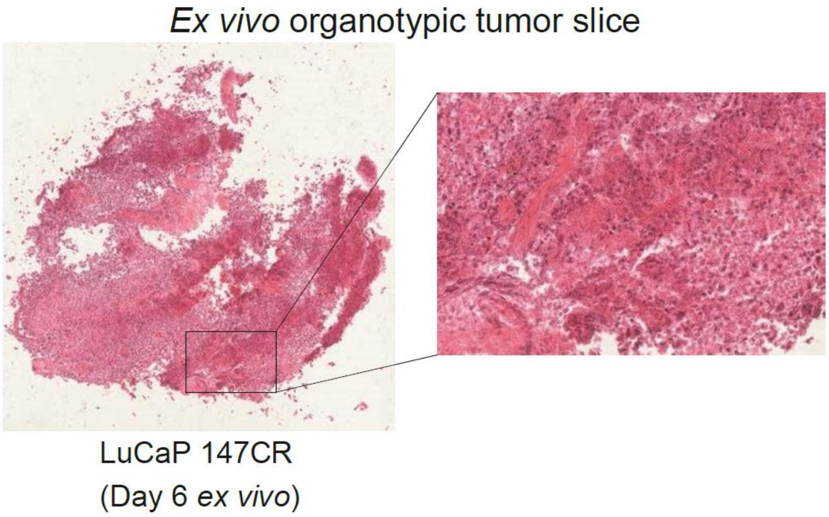

Figure S1. Organotypic tumor slice from an independent PDX tumor (147 CR).

Table S1. Characteristics of 42 LuCAP PDX tumor samples. NE denotes neuroendocrine; DNPC denotes double negative prostate cancer and CRPC denotes castration resistant prostate cancer.

Sample	Histology	LuCaP	Tissue implanted
1	NE	145.1	Liver met
2	NE	145.1	Liver met
3	NE	145.1	Liver met
4	NE	145.2	LN met
5	NE	145.2	LN met
6	NE	145.2	LN met
7	NE	93	TURP
8	NE	93	TURP
9	NE	93	TURP
10	NE	173.1	Liver met
11	NE	173.1	Liver met
12	NE	173.1	Liver met
13	DNPC	173.2	Rib met
14	DNPC	173.2	Rib met
15	DNPC	173.2	Rib met
16	CR	35CR	LN met
17	CR	35CR	LN met
18	CR	35CR	LN met
19	CR	70CR	Liver met
20	CR	70CR	Liver met
21	CR	70CR	Liver met
22	CR	73CR	Primary

23	CR	73CR	Primary
24	CR	73CR	Primary
25	CR	78CR	LN met
26	CR	78CR	LN met
27	CR	78CR	LN met
28	CR	86.2CR	Bladder met
29	CR	86.2CR	Bladder met
30	CR	86.2CR	Bladder met
31	CR	96CR	TURP
32	CR	96CR	TURP
33	CR	96CR	TURP
34	CR	105CR	Rib met
35	CR	105CR	Rib met
36	CR	105CR	Rib met
37	CR	147CR	Liver met
38	CR	147CR	Liver met
39	CR	147CR	Liver met
40	CR	167CR	Liver met
41	CR	167CR	Liver met
42	CR	167CR	Liver met

Table S2. List of validated antibodies used for RPPA analysis.

Unique ID	Specificity	Vendor	Catalog #
1	Phospho-S6 Ribosomal Protein (S235/236)	Cell Signaling Technology	2211
2	Phospho-S6 Ribosomal Protein (S240/244)	Cell Signaling Technology	2215
3	β-Catenin (6B3)	Cell Signaling Technology	9582
4	Phospho-cdc2 (Y15)	Cell Signaling Technology	9111
5	Phospho-Met (Y1349)	Cell Signaling Technology	3133
6	Phospho-Stat1 (Y701)	Cell Signaling Technology	9167
7	Phospho-MEK1/2 (S221) (166F8)	Cell Signaling Technology	2338
8	Phospho-GSK-3β (S9) (D85E12) XP®	Cell Signaling Technology	5558
9	Phospho-Src Family (Y416)	Cell Signaling Technology	2101
10	Phospho-p44/42 MAPK (Erk1/2) (T202/Y204)	Cell Signaling Technology	4377
11	Phospho-Gab1 (Y627)	Cell Signaling Technology	3233
12	Phospho-eIF4E (S209)	Cell Signaling Technology	9741
13	Phospho-cPLA2 (S505)	Cell Signaling Technology	2831
14	Phospho-Akt (S473) (193H12)	Cell Signaling Technology	4058
15	Phospho-Stat3 (Y705)	Cell Signaling Technology	9145
16	Phospho-CREB (S133)	Cell Signaling Technology	9198
17	Estrogen Receptor α	Cell Signaling Technology	2512
18	Phospho-PKC (pan) (βII S660)	Cell Signaling Technology	9371
19	Phospho-c-Jun (T91)	Cell Signaling Technology	2303
20	EpCAM (D4K8R) XP®	Cell Signaling Technology	36746
21	Phospho-Rac1/cdc42 (S71)	Cell Signaling Technology	2461
22	LDHA (C4B5)	Cell Signaling Technology	3582
23	p-NF-KB p65 (S536)	Cell Signaling Technology	3033
24	Phospho-Myosin Light Chain 2 (S19)	Cell Signaling Technology	3671
25	Phospho-ATF-2 (T71)	Cell Signaling Technology	9221
26	Phospho-RSK3 (T356/S360)	Cell Signaling Technology	9348
27	Phospho-MARCKS (S152/156)	Cell Signaling Technology	2741
28	p-SAPK/ JNK (T183/ Y185)	Cell Signaling Technology	9251
29	PAK1	Cell Signaling Technology	2602
30	Phospho-PAK1 (S199/204)/PAK2 (S192/197)	Cell Signaling Technology	2605
31	Phospho-c-Jun (T93)	Cell Signaling Technology	2993
32	Phospho-Paxillin (Tyr118)	Cell Signaling Technology	2541
33	PKM2 (D78A4) XP®	Cell Signaling Technology	4053
34	Phospho-EGFR(Y845)	Cell Signaling Technology	2231

35	Cleaved Caspase-7 (D198)	Cell Signaling Technology	9491
36	Phospho-HSP27 (S82)	Cell Signaling Technology	2401
37	Phospho-c-Jun (S63) II	Cell Signaling Technology	9261
38	Slug (C19G7) Rabbit	Cell Signaling Technology	9585
39	Phospho-eIF4G (S1108)	Cell Signaling Technology	2441
40	p-p38 (T180/ Y182)	Cell Signaling Technology	9215
41	FoxC1 (D8A6)	Cell Signaling Technology	8758
42	p-MDM2 (S166)	Cell Signaling Technology	3521
43	Phospho-YAP (S127) (D9W2I)	Cell Signaling Technology	13008
44	Phospho-Glycogen Synthase (Ser641)	Cell Signaling Technology	3891
45	Keratin 17/19 (D4G2) XP®	Cell Signaling Technology	12434
46	Snail (C15D3)	Cell Signaling Technology	3879
47	PKM1/2 (C103A3)	Cell Signaling Technology	3190
48	p-H2AX (S139)	Cell Signaling Technology	9718
49	c-Myc (D3N8F)	Cell Signaling Technology	13987
50	Phospho-Stat1 (Y701)	Cell Signaling Technology	9171
51	p-Rb (S807/ S811)	Cell Signaling Technology	9308
52	p-NF-KB p65 (S536)	Cell Signaling Technology	3033
53	p-H2AX (S139)	Cell Signaling Technology	9718
54	TCF8/ZEB1 (D80D3)	Cell Signaling Technology	3396
55	p-Akt 1/2/3 (S473)	Cell Signaling Technology	9271
56	p-SAPK/ JNK (T183/ Y185)	Cell Signaling Technology	9251
57	p-BCL-2 (S70)	Cell Signaling Technology	2827
58	BID	Cell Signaling Technology	2002
59	p-p53 (S20)	Cell Signaling Technology	9287
60	p-JNK (T183/ Y185)	Cell Signaling Technology	4671
61	p53	Cell Signaling Technology	2527
62	p-Chk1 (S345)	Cell Signaling Technology	2348
63	p-BAD (S112)	Cell Signaling Technology	5284
64	P-P53 (S15)	Cell Signaling Technology	9284
65	c-Caspase 3 (D175)	Cell Signaling Technology	9661
66	c-Src Antibody (N-16)	Santa Cruz Biotechnology	SC-19
67	N-Cadherin Antibody	Cell Signaling Technology	4061
68	Phospho-CaM I (S81)	Santa Cruz Biotechnology	SC-17019
69	Phospho-EGFR (Y1173)	Cell Signaling Technology	4407
70	p-EGFR Antibody (Y845)	Santa Cruz Biotechnology	SC-23420-R
71	ATM (D2E2)	Cell Signaling Technology	2873B
72	EphB4 Antibody (H-200)	Santa Cruz Biotechnology	SC-5536
73	phospho-(S/T) AMT/ATR Substrate	Cell Signaling Technology	2851B
74	EphA3 Antibody (H-80)	Santa Cruz Biotechnology	SC-25456
75	phospho-ATR (S428)	Cell Signaling Technology	2853
76	EphA4 Antibody (A-20)	Santa Cruz Biotechnology	SC-922
77	p-BRCA1 (S1524)	Cell Signaling Technology	9009
78	c-Myc Antibody (A-14)	Santa Cruz Biotechnology	SC-789
79	BRCA1 Antibody	Cell Signaling Technology	9010
80	phospho-ATM (S1981) (10H11.E12)	Cell Signaling Technology	4526
81	Pyruvate Dehydrogenase (C54G1)	Cell Signaling Technology	3205
82	Enolase-1 Antibody	Cell Signaling Technology	3810P
83	E-Cadherin (24E10)	Cell Signaling Technology	3195
84	Enolase-2 (D20H2)	Cell Signaling Technology	8171P
85	PFKFB2 (D7G5R)	Cell Signaling Technology	13045P
86	Androgen Receptor	Genetex/ Abcam	EPR1535
87	PFKFB3 (D7H4Q)	Cell Signaling Technology	13123P
88	DDR1 Antibody (C-20)	Santa Cruz Biotechnology	SC-532
89	Hexokinase II (C64G5)	Cell Signaling Technology	2867P
90	DDR2 Antibody (H-108)	Santa Cruz Biotechnology	SC-8989
91	PFKL Antibody	Cell Signaling Technology	8175P
92	EphA1 Antibody (G-18)	Santa Cruz Biotechnology	SC-925

93	Aldolase A (D73H4)	Cell Signaling Technology	8060
94	Trk C Antibody (798)	Santa Cruz Biotechnology	SC-117
95	PDHK1 (C47H1)	Cell Signaling Technology	3820P
96	EphA7 Antibody (C-19)	Santa Cruz Biotechnology	SC-918

Table S3. Top 25 Enrichr NEPC GO Biological Processes Results.

**Enrichr NEPC GO Biological Processes**	
glycolytic process	$p = 2.74 \times 10^{-8}$
carbohydrate catabolic process	$p = 7.97 \times 10^{-8}$
pyruvate metabolic process	$p = 1.96 \times 10^{-7}$
canonical glycolysis	$p = 1.38 \times 10^{-5}$
glucose catabolic process to pyruvate	$p = 1.38 \times 10^{-5}$
glycolytic process through glucose-6-phosphate	$p = 1.50 \times 10^{-5}$
positive regulation of cytoplasmic translation	$p = 0.001749$
negative regulation of cell-cell adhesion mediated by cadherin	$p = 0.002498$
regulation of cytoplasmic translation	$p = 0.003745$
neurotrophin TRK receptor signaling pathway	$p = 0.003745$
positive regulation of sprouting angiogenesis	$p = 0.003745$
apoptotic DNA fragmentation	$p = 0.003994$
positive regulation of stem cell proliferation	$p = 0.004243$
regulation of stem cell proliferation	$p = 0.004243$
positive regulation of amyloid-beta formation	$p = 0.004492$
cellular response to alkaloid	$p = 0.004492$
regulation of cell-cell adhesion mediated by cadherin	$p = 0.004741$
neurotrophin signaling pathway	$p = 0.005239$
extrinsic apoptotic signaling pathway in absence of ligand	$p = 0.005239$
positive regulation of amyloid precursor protein catabolic process	$p = 0.005488$
regulation of vacuole organization	$p = 0.005488$
DNA catabolic process, endonucleolytic	$p = 0.005737$
apoptotic nuclear changes	$p = 0.005986$
hippo signaling	$p = 0.006484$
regulation of sprouting angiogenesis	$p = 0.008224$

Table S4. Top 25 Enrichr NEPC Bioplanet Results.

**Enrichr NEPC Bioplanet**	
Glycolysis and gluconeogenesis	$p = 5.04 \times 10^{-7}$
Pyruvate metabolism	$p = 6.60 \times 10^{-5}$
HIF-1 transcriptional activity in hypoxia	$p = 1.07 \times 10^{-4}$
NADE-dependent death signaling	$p = 0.001499$
Apoptotic factor-mediated response	$p = 0.001749$
TSP1-induced apoptosis in microvascular endothelial cell	$p = 0.001999$
West Nile virus infection	$p = 0.002248$
Apoptotic DNA fragmentation and tissue homeostasis	$p = 0.002498$
DCC role in regulating apoptosis	$p = 0.002498$
Apoptotic cleavage of cell adhesion proteins	$p = 0.002747$
Caspase-mediated cleavage of cytoskeletal proteins	$p = 0.002997$
D4-GDI signaling pathway	$p = 0.002997$
Granzyme A-mediated apoptosis pathway	$p = 0.002997$
Activation of DNA fragmentation factor	$p = 0.003246$
Stress induction of HSP regulation	$p = 0.003745$
Hypoxia-inducible factor in the cardivascular system	$p = 0.003994$
B cell survival pathway	$p = 0.003994$
Apoptotic cleavage of cellular proteins	$p = 0.004243$
Metabolism	$p = 0.004641$
Apoptosis modulation by HSP70	$p = 0.004741$
Cell cycle progression regulation by PLK3	$p = 0.004741$

Apoptotic signaling in response to DNA damage	$p = 0.00499$
Signaling by Hippo	$p = 0.00499$
Mitochondrial role in apoptotic signaling	$p = 0.005239$
Glycolysis	$p = 0.006732$

Table S5. Top 25 Enrichr CRPC GO Biological Processes Results.

**Enrichr CRPC GO Biological Processes**	
response to indole-3-methanol	$p = 2.75 \times 10^{-6}$
cellular response to indole-3-methanol	$p = 2.75 \times 10^{-6}$
entry of bacterium into host cell	$p = 7.69 \times 10^{-6}$
cellular response to organonitrogen compound	$p = 2.12 \times 10^{-5}$
entry into host	$p = 2.14 \times 10^{-5}$
positive regulation of transferase activity	$p = 6.27 \times 10^{-5}$
cellular response to organic cyclic compound	$p = 6.53 \times 10^{-5}$
positive regulation of neuron apoptotic process	$p = 7.54 \times 10^{-5}$
cellular response to growth factor stimulus	$p = 7.62 \times 10^{-5}$
positive regulation of nucleic acid-templated transcription	$p = 1.20 \times 10^{-4}$
cellular response to vascular endothelial growth factor stimulus	$p = 1.53 \times 10^{-4}$
response to estrogen	$p = 1.62 \times 10^{-4}$
regulation of cell-cell adhesion	$p = 2.45 \times 10^{-4}$
positive regulation of DNA-binding transcription factor activity	$p = 2.82 \times 10^{-4}$
positive regulation of neuron death	$p = 2.93 \times 10^{-4}$
cellular response to alcohol	$p = 3.06 \times 10^{-4}$
positive regulation of phosphorylation	$p = 3.06 \times 10^{-4}$
adherens junction organization	$p = 3.19 \times 10^{-4}$
regulation of DNA-templated transcription, initiation	$p = 4.94 \times 10^{-4}$
regulation of apoptotic process	$p = 5.03 \times 10^{-4}$
regulation of transcription initiation from RNA polymerase II promoter	$p = 5.44 \times 10^{-4}$
regulation of extrinsic apoptotic signaling pathway	$p = 5.44 \times 10^{-4}$
positive regulation of apoptotic process	$p = 5.55 \times 10^{-4}$
regulation of cell population proliferation	$p = 5.62 \times 10^{-4}$
positive regulation of type I interferon production	$p = 7.87 \times 10^{-4}$

Table S6. Top 25 Enrichr CRPC Bioplant Results.

**Enrichr CRPC Bioplant**	
Nuclear beta-catenin signaling and target gene transcription regulation	$p = 9.94 \times 10^{-6}$
Androgen receptor signaling, proteolysis, and transcription regulation	$p = 1.32 \times 10^{-5}$
Prostate cancer	$p = 1.37 \times 10^{-5}$
Apoptotic cleavage of cell adhesion proteins	$p = 1.51 \times 10^{-5}$
Pathways in cancer	$p = 2.06 \times 10^{-5}$
Cell to cell adhesion signaling	$p = 3.72 \times 10^{-5}$
Downregulation of MTA-3 in ER-negative breast tumors	$p = 4.19 \times 10^{-5}$
E-cadherin keratinocyte pathway	$p = 5.74 \times 10^{-5}$
Integrated breast cancer pathway	$p = 6.79 \times 10^{-5}$
Adherens junction actin cytoskeletal organization	$p = 1.11 \times 10^{-4}$
Thyroid cancer	$p = 1.11 \times 10^{-4}$
Inactivation of GSK3 by Akt causes accumulation of beta-catenin in alveolar macrophages	$p = 1.19 \times 10^{-4}$
Nectin adhesion pathway	$p = 1.19 \times 10^{-4}$
Interleukin-2/PI3K pathway	$p = 1.62 \times 10^{-4}$
Trefoil factor initiation of mucosal healing	$p = 1.91 \times 10^{-4}$
p38 alpha/beta MAPK downstream pathway	$p = 1.91 \times 10^{-4}$
E-cadherin nascent AJ-like junctions pathway	$p = 2.02 \times 10^{-4}$
TWEAK signaling pathway	$p = 2.34 \times 10^{-4}$
Apoptosis	$p = 2.69 \times 10^{-4}$
Post-translational regulation of adherens junction stability and disassembly	$p = 3.06 \times 10^{-4}$
Arf6 integrin-mediated signaling pathway	$p = 3.19 \times 10^{-4}$

BDNF signaling pathway	$p = 3.35 \times 10^{-4}$
Apoptotic execution phase	$p = 3.59 \times 10^{-4}$
Endometrial cancer	$p = 3.59 \times 10^{-4}$
Pathogenic Escherichia coli infection	$p = 4.32 \times 10^{-4}$

© 2022 by the authors. Licensee MDPI, Basel, Switzerland. This article is an open access article distributed under the terms and conditions of the Creative Commons Attribution (CC BY) license (http://creativecommons.org/licenses/by/4.0/).
